# Who is More Likely (Not) to Make Home-Based Work Trips During the COVID-19 Pandemic? The Case of Scotland

**DOI:** 10.1177/03611981221119192

**Published:** 2022-09-20

**Authors:** Torran Semple, Grigorios Fountas, Achille Fonzone

**Affiliations:** 1 School of Computer Science, University of Nottingham, Nottingham, UK; 2 Department of Transportation and Hydraulic Engineering, School of Rural and Surveying Engineering, Aristotle University of Thessaloniki, Thessaloniki, Greece; 3 School of Engineering and The Built Environment, Transport Research Institute, Edinburgh Napier University, Edinburgh, UK

**Keywords:** COVID-19, Work trips, Telecommuting, Infection risk, Zero-inflated hierarchical ordered probit

## Abstract

In this study, we used survey data (*n* = 6,000) to investigate the work trip patterns of Scottish residents at various points of the COVID-19 pandemic. We focused specifically on the reported patterns of weekly work trips made during the government-enforced lockdown and subsequent phases of restriction easing. This was of particular importance given the widespread changes in work trips prompted by COVID-19, including a significant rise in telecommuting and a reduction in public transport commuting trips. The survey data showed that the vast majority of respondents (∼85%) made no work trips during lockdown, dropping to ∼77% following the easing of some work-related restrictions. Zero-inflated hierarchical ordered probit models were estimated to determine the sociodemographic and behavioral factors affecting the frequency of work trips made during three distinct periods. The model estimation results showed that the socioeconomic characteristics of respondents influenced work trips made throughout the pandemic. In particular, respondents in households whose main income earner was employed in a managerial/professional occupation were significantly more likely to make no work trips at all stages of the pandemic. Those with a health problem or disability were also significantly more likely to make no work trips throughout the pandemic. Other interesting findings concern respondents’ gender, as males were more likely to complete frequent work trips than females throughout the pandemic, and differences between densely populated areas and the rest of Scotland, as respondents from a large city (Edinburgh or Glasgow) were significantly more likely to make frequent work trips as restrictions were eased.

The COVID-19 pandemic, and subsequent government-enforced lockdowns, have drastically affected the travel behavior of many people across the world. Widespread changes in mode preferences were recorded, while trip purposes also varied significantly from pre-COVID-19 norms (*1* [Bibr bibr2-03611981221119192]–*3*). In the UK, the most stringent lockdown was introduced in March 2020. During this period, all nonessential travel was prohibited and residents were advised to work from home (i.e., telecommuting) unless they were a “key worker” (healthcare, social care, essential shops, etc.) (*4*). The UK Government also introduced a furlough scheme in March 2020, providing temporary financial support for those in occupations that could not be completed from home. National statistics during April 2020 allow the scale of altered commuting behavior to be appreciated; the Office for National Statistics (ONS) found that 46.6% of British residents in full- or part-time employment telecommuted during lockdown, and 86.0% of those who telecommuted did so as a result of COVID-19 (*5*). Given that these restrictions altered the commuting patterns of many British residents, it is important that the sociodemographic and habitual factors affecting COVID-19 work trips are understood. This is particularly important in the context of public health, as those who continued to work (in the workplace) during the pandemic were at elevated risk of contracting COVID-19 compared with the rest of the population (*6*). Furthermore, ONS found that ethnic minority groups and those living in deprived areas were significantly more likely to contract COVID-19 than those from white ethnic backgrounds and those living in affluent areas (*7*). Given the link between continued work throughout the pandemic and higher COVID-19 infection risk, it is important to better understand the COVID-19 commuting behavior of these groups.

Recent studies across Europe show that the COVID-19 pandemic has caused a significant reduction in public transport usage. This reduction is deemed to be the combined result of a rise in telecommuting, how risky the public perceive public transport to be in relation to COVID-19 contraction, and the restrictions placed on public transport capacity (*8*, *9*). Studies investigating public transport travel intentions have found that decreases in public transport usage may persist after the pandemic (*2*). It has also been found that some workers intend to telecommute more following the pandemic (*10*, *11*). In this study, the work trip patterns of Scottish residents during various distinct periods of the COVID-19 pandemic are explored via a statistical analysis of survey data. Specifically, discrete outcome modeling approaches are utilized to determine the sociodemographic and behavioral factors affecting the frequency of work trips made by Scottish residents. The analysis of work trips provides insights into the groups who have been more negatively affected by the pandemic, with respect to disproportionate infection exposure related to the inability to complete work remotely. Our results may also provide useful insights into the potential legacy of COVID-19 commuting behaviors, such as increased telecommuting and decreased public transport usage. Such commuting trends are likely to have a significant impact on transport policy and planning in the coming years.

## Data

The data used for the statistical analysis were obtained from Transport Scotland’s triweekly COVID-19 Public Attitudes Survey (*n* = 6,000), conducted from March 2020 to October 2020. Transport Scotland employed a consultancy to conduct repeated cross-sectional survey waves. Nine survey waves were available for the current research. The samples for each wave were drawn from Scottish postcodes, which were randomly selected to fairly represent the Scottish Index of Multiple Deprivation (SIMD) regional quotas. SIMD is a standardized approach for ranking deprivation in Scottish subregions. SIMD considers multiple deprivation indicators, including income, employment status, education levels, access to health services, crime rates, and housing quality (*12*). The survey waves were conducted telephonically (landline and mobile) and were subject to the General Data Protection Regulation (GDPR) and Market Research Society (MRS) Code of Conduct. The MRS Code of Conduct provides ethical and professional standards, based mostly on aspects of the GDPR, that research practitioners must uphold (*13*). The telephone numbers (80% landline and 20% mobile) were chosen randomly from the households with a landline in the selected postcode areas. Numbers classified as “nonresponse”, “business premises”, or “refusal to participate” were discarded. According to the UK Government Office for Communications (Ofcom), 66% of Scottish households have landlines, as a result, the remaining 34% of households that do not have a landline are not accounted for in the survey sample (*14*). Furthermore, Ofcom data show that landline ownership is lower among young people and students, therefore, a considerable proportion of these individuals would have been difficult to reach.

The surveys aimed to provide insights into the COVID-19 travel behavior of Scottish residents, as well as exploring future travel intentions. The surveys also recorded sociodemographic (e.g., gender, ethnic background, disability, location, and household social grade based on the occupation of the household’s main income earner) and behavioral (e.g., preferred mode of travel before and during the pandemic, and other reactive behaviors to COVID-19) characteristics of respondents. For an extensive list of all available independent variables, see Appendix: Table A1. “Household social grade” is defined by the Scottish Government as follows: social AB households are those whose main income earner is employed in a managerial/professional occupation; social C1 households’ main earner is in a supervisory/junior managerial occupation or in full-time education; social C2 households’ main earner is in a skilled manual occupation; and social DE households’ main earner is in a semiskilled/unskilled manual occupation or is unemployed (*15*). The survey samples were almost exactly representative of Scotland’s demographic strata, particularly in relation to gender, age, household social grade, ethnic background, region of Scotland, and health problems or disabilities.

We studied the weekly work trips of Scottish residents during three distinct periods of the pandemic, as shown in Table 1. The survey waves were aggregated based on the period in which they were conducted. These periods are defined with respect to the Scottish Government’s “COVID-19 route map,” which presented the phased easing of lockdown restrictions. The aggregated survey waves can be contextualized as follows: the “Lockdown” sample contains two survey waves conducted during the most stringent lockdown period; the “Phases 1 to 2” sample includes two survey waves conducted during Phases 1 and 2 of the Scottish Government’s route map; and the “Phase 3” sample includes five survey waves conducted during Phase 3 of the route map. The most pertinent restrictions during each stage of the route map were as follows: Lockdown prohibited all nonessential work, prompting widespread telecommuting and the UK Government’s furlough scheme; Phase 1 included a slight relaxation of working restrictions, allowing those who could not complete their work from home, and who were also able to complete their jobs outside in a socially distanced manner (e.g., builders and other forms of outdoor labor), to return to their workplace; Phase 2 included further incremental easing of working restrictions; and Phase 3 included the reopening of many nonessential workplaces and businesses, including food outlets, clothes shops, and gyms (*16*).

**Table 1.** Aggregation of Survey Waves Based on the Scottish Government’s COVID-19 Route Map

Route map (Lockdown/Phase)	Survey waves
Lockdown (March 24–May 27, 2020)	Wave 1 (May 5–13, 2020)
	Wave 2 (May 18–25, 2020)
Phase 1 (May 28–June 17, 2020)	Wave 3 (June 1–7, 2020)
Phase 2 (June 18–July 8, 2020)	Wave 4 (June 24–27, 2020)
Phase 3 (July 9–October 8, 2020)	Wave 5 (July 8–13, 2020)
	Wave 6 (July 22–28, 2020)
	Wave 7 (August 19–25, 2020)
	Wave 8 (September 8–16, 2020)
	Wave 9 (September 30–October 6, 2020)

The question corresponding to our dependent variable was, “In the past 7 days, how many times have you left your home to go to work?” It should be noted that the survey question refers to home-based work trips, which excludes certain other trips that could be perceived as work trips, for example, if someone were to travel between offices for meetings. Responses were recorded using an ordinal scale with six possible outcomes (zero, one, two to three, four to five, six to seven, or more than seven trips). Owing to the small number of responses for some of these categories (less than 30 responses), the levels of the dependent variable were aggregated as follows: Level 0: no trips, Level 1: 1 to 3 trips, Level 2: 4 to 5 trips, and Level 3: 6 or more trips. Kolmogorov–Smirnov tests were conducted to test for the presence of significant variation across distributions of responses for grouped survey waves (for example, between Wave 1 and Wave 2 for the Lockdown sample). All tests produced statistically insignificant results, therefore there was no significant variation in the distributions of the survey waves within each sample. Further Kolmogorov–Smirnov tests were conducted between the samples. Two tests produced significant results, indicating that the distribution of work trips made by the Lockdown and Phase 3 respondents were significantly different, which was also the case between Phases 1 to 2 and Phase 3 respondents. There was, however, no significant variation in the distribution of work trips made by Lockdown and Phases 1 to 2 respondents, which is likely to be attributable to the relatively minor easing of commuting restrictions during Phases 1 and 2.

**Figure 1.**
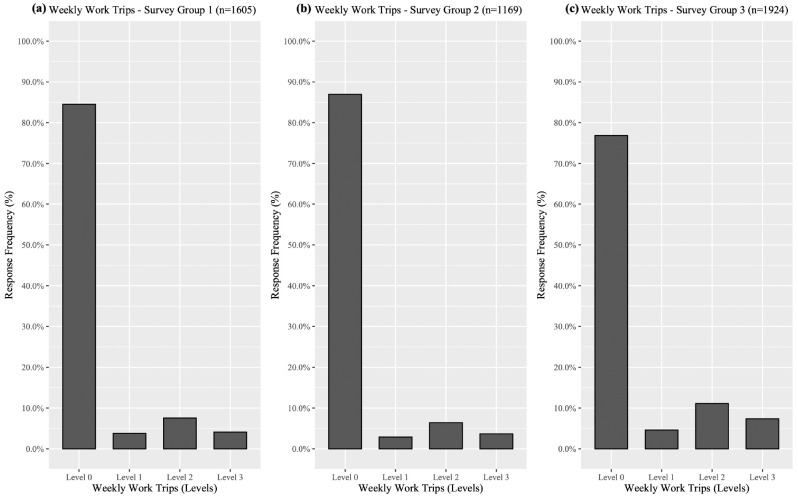
Distribution of weekly work trips made by Scottish residents during (*a*) Lockdown (Survey Group 1), (*b*) Phases 1 to 2 (Survey Group 2), and (*c*) Phase 3 (Survey Group 3), in which Level 0 = no trips, Level 1 = one to three trips, Level 2 = four to five trips, and Level 3 = six or more trips.

Figure 1 shows the distribution of weekly work trips made by respondents per survey sample (*n* = 4698 overall for the three groups, following omission of incomplete observations). A majority of respondents indicated that they made no weekly work trips during all stages of the pandemic, however, a larger proportion of Phase 3 respondents (∼23%) made at least one weekly work trip. The substantial number of observations related to the lowest outcome of the dependent variable is known as zero-inflation. From a statistical perspective, this mobility pattern leads to a clustering of work trip responses around the zero-frequency level. To effectively account for the preponderance of zero-frequency responses, an adaptation of the ordered probit modeling framework, the zero-inflated hierarchical ordered probit (ZIHOP), was adopted to analyze the frequency of work trips made throughout the pandemic.

During lockdown, COVID-19 restrictions prevented all nonessential work, therefore the proportion of respondents making at least one weekly work trip during this period (∼15%) was likely to have been key workers. Working restrictions were similar during Phases 1 to 2, with the only difference being a return to work for some manual laborers who could complete most of their work outdoors. Despite this, only ∼13% of the Phases 1 to 2 sample made at least one weekly work trip, a marginal decrease compared with Lockdown. The slight increase in work trips among Phase 3 respondents may be the result of nonessential shops, restaurants, and other leisure facilities reopening during Phase 3. It should be noted that owing to the formulation of the survey, Level 0 of the dependent variable includes people who did not make work trips before the pandemic, for example, students, pensioners, those who worked from home previously, and those who were unemployed. In 2019, 3.5% of the economically active (aged over 16 and able to work) Scottish population were unemployed (*17*). Furthermore, approximately 16% of the Scottish population worked from home in 2019 (*18*).

## Methodology

The dependent variables, representing trip frequencies among respondents, were recorded as discrete, ordered outcomes, therefore, the ordered probit modeling framework was deemed appropriate for the statistical analysis (*19*).

As discussed in the section, “Data,” the distributions of work trips across the survey periods were zero-inflated, therefore, the methodological approach was altered to account for this characteristic. The zero-inflated ordered probit (ZIOP) approach has an improved capacity to cater for distributions of ordinal variables with overrepresentation of the zero outcome, compared with the standard ordered probit and related approaches (*20*–*23*). For this research, the ZIOP framework, in particular the ZIHOP, were adopted to account for the zero-inflated distribution of work trips. The ZIHOP differs from the ZIOP such that thresholds are allowed to vary across observations, therefore accounting for threshold heterogeneity. The ZIOP framework consists of two distinct processes: firstly, a binary probit process, which assigns outcomes between the zero-state and the ordered state of trip frequencies and, secondly, an ordered probit process, which estimates the impacts of the observed determinants of the ordered outcomes under the condition that they do not belong to the zero-state (*19*). In other words, the ZIOP framework allows two behavioral mechanisms to be captured simultaneously, such that “structural zeros” and “probabilistic zeros” can be modeled in one framework. In this study, (i) structural zeros indicate respondents who usually did not travel for work, whereas (ii) probabilistic zeros indicate respondents who probably traveled for work in general but had not in the previous 7 days. On the basis of this distinction for zero observations, the zero-state corresponds to structural zeros, whereas the ordered state includes probabilistic zeros as well as nonzero observations (which capture respondents who had traveled at least once in the previous 7 days). In this context, the ZIOP models will not only unveil the factors affecting the likelihood for various trip frequencies, but will also help identify the factors determining the likelihood of a zero-frequency response belonging either to the zero- or the ordered state.

The ZIOP framework consists of two latent variable equations, which correspond to the binary and ordered probit processes. The splitting function, between the zero-state and ordered state, is expressed through a binary probit model,

(1) $$k_{n}^{*}=dC_{n}+\omega_{n},k_{n}=0\;\mathrm{if}\;k_{n}^{*}\leq 0,k_{n}=1\;\mathrm{otherwise}$$

where

$k_{n}^{*}$ is a continuous latent variable, whose observed counterpart is a binary variable $k_{n}$ , with $k_{n}$ representing whether an outcome belongs to the zero-state $\left(k_{n}=0\right)$ or the ordered state $\left(k_{n}=1\right)$ ;

C represents a vector of independent variables, which influence whether an outcome belongs to the zero or ordered state;

d is a vector of estimable parameters; and

$\omega_{n}$ is the disturbance term, assumed to be normally distributed with mean = 0 and variance = 1.

The probability that an outcome belongs to the ordered state is calculated as follows:

(2) $$P_{n}\left(k_{n}=1\right)=\Phi (dC_{n})$$

where Φ denotes the standardized cumulative normal distribution. In turn, the probability of an outcome belonging to the zero-state is equal to $1-P_{n}\left(k_{n}=1\right)$ . The ordered state can be defined by means of another latent variable, $z_{n}^{*}$ , similar to $k_{n}^{*}$ ,

(3) $$z_{n}^{*}=\beta X_{n}+\varepsilon_{n}$$

where

β is a vector of estimable parameters;

**X** is a vector of explanatory variables influencing the discrete ordered outcome for an observation, *n*; and

*ε* is random disturbance, assumed to be normally distributed across observations, with mean = 0 and variance = 1.

Finally,

(4) $$z_{n}=\left\{ \begin{array}{cc} 0 & \mathrm{if}\;z_{n}^{*}\leq \mu_{0}=0\;\mathrm{or}\;k_{n}=0\\ j & \mathrm{if}\;\mu_{j-1}<z_{n}^{*}\leq \mu_{j}\;\mathrm{and}\;k_{n}=1,\;\mathrm{for}\;1\leq j<J\\ J & \mathrm{if}\;z_{n}^{*}>\mu_{J-1}\;\mathrm{and}\;k_{n}=1 \end{array}\right.$$

where

$z_{n}$ is an integer that corresponds to an observed outcome of a given observation, *n*;

*j* denotes the observed outcome;

*J* denotes the highest order outcome (Level 3, six or more trips, in our case); and

µ are threshold parameters distinguishing the ordered outcomes.

It should be noted that $\mu_{0}$ is conventionally equal to zero, as shown in the first line of Equation 4, therefore, only J−2 thresholds need to be estimated (*19*). The conditional probabilities corresponding to various ordered outcomes, given that the observation belongs to the ordered state, are expressed as follows:

(5) $$P_{n}\left(z_{n}=0|k_{n}=1\right)=\Phi (-\beta X_{n})$$

(6) $$P_{n}\left(z_{n}=j|k_{n}=1\right)=\Phi \left(\mu_{j}-\beta X_{n}\right)-\Phi \left({\mu_{j}}_{-1}-\beta X_{n}\right)\;\mathrm{for}\leq j<J$$

(7) $$P_{n}\left(z_{n}=J|k_{n}=1\right)=1-\Phi \left(\mu_{J-1}-\beta X_{n}\right),$$

from which the unconditional probabilities of a given outcome are

(8) $$P_{n}\left(z_{n}=0\right)=P_{n}\left(k_{n}=0\right)+P_{n}\left(z_{n}=0|k_{n}=1\right)\cdot \;P_{n}\left(k_{n}=1\right)$$

(9) $$P_{n}\left(z_{n}=j\right)=P_{n}\left(z_{n}=j|k_{n}=1\right)\;\cdot \;P_{n}\left(k_{n}=1\right),1\leq j\leq J$$

As discussed previously, the advantage of the ZIHOP over the ZIOP, is that threshold heterogeneity may be explicitly accounted for (*20*). To fulfill this, thresholds are allowed to vary across observations, such that,

(10) $$\mu_{n,j}=\exp(t_{j}+\gamma S_{n})$$

where

**S** are vectors of variables influencing the thresholds,

γ are vectors of estimable parameters for **S**, and

$t_{j}$ is the threshold intercept term.

To identify the relative impact of the independent variables on the probabilities of the ordered outcomes, as well as on the probability of an observation belonging to the zero- or ordered state, marginal effects were also computed, following the approach suggested by Fountas and Anastasopoulos (*21*). Following model estimation, goodness-of-fit (GOF) and statistical fit metrics were used to compare competing models. The Akaike information criterion (AIC) and the likelihood ratio test (LRT), which evaluate GOF and statistical fit (*19*), respectively, were the primary means of model evaluation. The ZIHOP models were estimated in NLOGIT 6 (*24*), whereas any other statistical testing or data visualization was performed in R.

## Model Estimation Results

Table 2 displays the descriptive statistics for the independent variables that were found to be influential in the model estimations. Table 3 shows the model estimations for weekly work trips made during Lockdown, Phases 1 to 2, and Phase 3, for which statistically significant variables were those with *t*-stats > 1.65 (corresponding to >90% level of confidence [LOC]) or *t*-stats > 1.96 (corresponding to >95% LOC). The McFadden’s *R*-squared values in Table 3 offer insights into the statistical fit of the models. It should be noted that although such fit was limited for the Lockdown and Phase 3 models, the models still provided valuable insights into the determinants of structural and probabilistic zeros. Table 4 displays the average marginal effects, which allow further insights into the effect of a given independent variable on the interior categories of the dependent variable (Level 1 [1 to 3 trips] and Level 2 [4 to 5 trips]), corresponding to the ordered outcomes of each survey period. The “Ordered state” column in Table 4 provides the marginal effects for the splitting function between the zero- and ordered state, for which positive effects are associated with increased likelihood of the ordered state. Significant correlated disturbances were found between the binary- and ordered probit components for the Phases 1 to 2 ZIHOP model, therefore, this is referred to as a zero-inflated hierarchical ordered probit model with correlated disturbances (ZIHOPCD). Please note that any instances of "na" in Table 3, Table 4 and Table 5 indicate than an independent variable was not included for a given model.

**Table 2.** Descriptive Statistics for Key Independent Variables in the Lockdown, Phases 1 to 2, and Phase 3 Models

Variable description	Lockdown (%)	Phases 1 to 2 (%)	Phase 3 (%)
Socioeconomic characteristics			
Household social grade (1 if managerial/professional occupation, 0 otherwise)	35.95	30.97	36.80
Household social grade (1 if semiskilled/unskilled manual occupation or unemployed, 0 otherwise)	20.31	20.87	19.28
Demographic characteristics			
Age indicator (1 if under 25, 0 otherwise)	14.14	14.37	14.19
Age indicator (1 if over 55, 0 otherwise)	36.01	38.32	38.10
Health problem or disability (1 if yes, 0 if no)	20.31	27.89	25.73
Gender (1 if male, 0 otherwise)	47.17	47.99	49.84
Ethnic background (1 if white British, 0 otherwise)	88.91	90.5	84.30
Region of Scotland (1 if major city [Edinburgh or Glasgow], 0 otherwise)	48.22	45.77	46.88
Directly affected by COVID-19 (1 if yes, 0 if no)	22.99	21.12	21.26
Behavioral characteristics			
Mode of travel before lockdown (1 if personal vehicle used frequently, 0 if not used frequently)	83.43	76.48	77.91

**Table 3.** ZIHOP/ZIHOPCD Model Estimations for Work Trips Made by the Lockdown, Phases 1–2, and Phase 3 Samples

Variable description	Lockdown ZIHOP model		Phases 1 to 2 ZIHOPCD model		Phase 3 ZIHOP model	
	Coefficient	*t*-Stat	Coefficient	*t*-Stat	Coefficient	*t*-Stat
Splitting function between ordered and zero-state						
Constant*	−0.490	−3.22	1.409	2.23	−0.237	−2.32
Household social grade (1 if managerial/professional occupation, 0 otherwise)	−0.334	−3.28	na	na	na	na
Household social grade (1 if semiskilled/unskilled manual occupation or unemployed, 0 otherwise)	−0.249	−1.94	na	na	na	na
Health problem or disability (1 if yes, 0 if no)	−0.876	−5.73	−1.389	−1.79	−0.901	−8.37
Directly affected by COVID-19 (1 if yes, 0 if no)†	na	na	0.588	1.09	na	na
Ordered state						
Constant	0.351	0.89	−0.872	−3.20	0.277	1.20
Mode of travel before lockdown (1 if personal vehicle used frequently, 0 if not used frequently)	0.384	1.68	0.276	2.07	0.566	3.94
Ethnic background (1 if White British background, 0 otherwise)	na	na	na	na	−0.456	−2.83
Gender (1 if male, 0 if female)	0.326	1.95	0.172	1.85	0.647	5.27
Age indicator (1 if over 55, 0 otherwise)	−0.700	−3.68	−0.402	−3.28	na	na
Region of Scotland (1 if major city [Edinburgh or Glasgow], 0 otherwise)	na	na	na	na	0.250	2.22
Household social grade (1 if managerial/professional occupation, 0 otherwise)	na	na	−0.215	−2.08	−0.383	−3.03
Household social grade (1 if semiskilled/unskilled manual occupation or unemployed, 0 otherwise)	na	na	na	na	−0.456	−2.74
Intercept for Threshold 1	−0.806	−2.52	−2.246	−8.92	−0.930	−5.00
Intercept for Threshold 2	0.336	1.67	−0.744	−3.14	0.315	2.83
Heterogeneity in threshold parameters (Threshold 1)						
Region of Scotland (1 if major city [Edinburgh or Glasgow], 0 otherwise)	0.214	2.04	0.594	3.33	na	na
Age indicator (1 if less than 25, 0 otherwise)	na	na	na	na	0.189	1.77
Correlation of disturbances						
Correlation coefficient	na	na	−0.763	−2.58	na	na
Number of observations	1,605		1,169		1,924	
Log-likelihood at zero, LL(0)	−992.87		−825.95		−1,537.01	
Log-likelihood at convergence, LL(**β**)	−905.45		−557.89		−1,420.92	
McFadden’s R-squared, 1- LL(**β**)/LL(0)	0.09		0.32		0.08	
AIC at zero	1,985.74		1,651.90		3,074.02	
AIC at convergence for ZIOP	1,835.55		1,150.16		2,867.2	
AIC at convergence for ZIHOP (ZIHOPCD for Phases 1 to 2 model)	1,832.91		1,139.78		2,865.9	
Likelihood ratio tests	(LRT I) ZIHOP>ZIOP with >97% LOC		(LRT II) ZIHOPCD>ZIOP with >99% LOC		(LRT III) ZIHOP>ZIOP with >93% LOC	

*Note*: ZIHOP = zero-inflated hierarchical ordered probit; ZIHOPCD = zero-inflated hierarchical ordered probit model with correlated disturbances; ZIOP = zero-inflated ordered probit; AIC = Akaike information criterion; LRT = likelihood ratio test.

* Some constant terms are statistically insignificant; however, they were retained in the final models given that n-2 thresholds were estimated (*19*). An insignificant constant term still captures the average effect of unobserved variables on the dependent variable (*25*).

† Given that the specific variable produced a low *t*-statistic, a likelihood ratio test (*19*) was carried out to further assess its statistical significance. The test results showed that the inclusion of the specific variable resulted in a significant improvement of the model fit (as indicated by the log-likelihood value), at a confidence level greater than 99%. Therefore the variable was retained in the model.

**Table 4.** Average Marginal Effects of Estimated Models

Variable description	Average marginal effects				
	Ordered state	Level 0 (no trips)	Level 1 (1 to 3 trips)	Level 2 (4 to 5 trips)	Level 3 (6 or more trips)
Lockdown model					
Household social grade (1 if managerial/professional occupation, 0 otherwise)	−0.090	na	na	na	na
Household social grade (1 if semiskilled/unskilled manual occupation or unemployed, 0 otherwise)	−0.066	na	na	na	na
Health problem or disability (1 if yes, 0 if no)	−0.192	na	na	na	na
Age indicator (1 if over 55, 0 otherwise)	na	0.245	0.028	−0.117	−0.157
Mode of travel before lockdown (1 if personal vehicle used frequently, 0 if not used frequently)	na	−0.138	−0.014	0.069	0.083
Gender (1 if male, 0 if female)	na	−0.110	−0.020	0.048	0.081
Phases 1 to 2 model					
Gender (1 if male, 0 if female)	na	−0.050	0.006	0.019	0.025
Age indicator (1 if over 55, 0 otherwise)	na	0.112	−0.013	−0.044	−0.055
Household social grade (1 if managerial/professional occupation, 0 otherwise)	na	0.060	−0.007	−0.024	−0.029
Health problem or disability (1 if yes, 0 if no)	−0.101	na	na	na	na
Mode of travel before lockdown (1 if personal vehicle used frequently, 0 if not used frequently)	na	−0.075	0.009	0.030	0.036
Directly affected by COVID-19 (1 if yes, 0 if no)	0.094	na	na	na	na
Phase 3 model					
Household social grade (1 if managerial/professional occupation, 0 otherwise)	na	0.135	0.016	−0.051	−0.100
Household social grade (1 if semiskilled/unskilled manual occupation or unemployed, 0 otherwise)	na	0.166	0.014	−0.070	−0.110
Gender (1 if male, 0 if female)	na	−0.223	−0.033	0.078	0.178
Mode of travel before lockdown (1 if personal vehicle used frequently, 0 if not used frequently)	na	−0.207	−0.016	0.089	0.134
Ethnic background (1 if white British background, 0 otherwise)	na	0.142	0.031	−0.032	−0.141
Region of Scotland (1 if major city [Edinburgh or Glasgow], 0 otherwise)	na	−0.086	−0.013	0.029	0.069
Health problem or disability (1 if yes, 0 if no)	−0.279	na	na	na	na

## Discussion of Results

A summary of the significant factors affecting work trips throughout the pandemic are displayed in Table 5. To facilitate the interpretation of the variables determining the splitting function between the ordered and zero-state, we provide the following example: the negative sign for the “household social grade (AB)” variable in Table 3 indicates that this group is significantly more likely than other social grades to belong to the zero-state (and, subsequently, less likely to belong to the ordered state), therefore, this variable is denoted by a downwards black arrow (“↓”) in Table 5. For the variables affecting the ordered state, a significantly positive independent variable increases the likelihood of frequent weekly work trips (six or more) (“↑”), whereas a significantly negative variable increases the likelihood of no work trips (“↓”). Other features in Table 5 can be interpreted as follows: for heterogeneity in threshold parameters (specifically for Threshold 1) “↓” and “↑” show that a given variable increases the likelihood of a response to Level 1 (1 to 3 trips) and Level 2 (4 to 5 trips), respectively; “na” indicates that a variable is not present within a given model; and the number of arrows indicates the strength of marginal effects associated with each independent variable (regardless of direction): (moderate) ↑ = 0.0000 to 0.0999; (strong) ↑↑ = 0.1000 to 0.1999; (very strong) ↑↑↑ >0.1999. The relative strengths of the marginal effects allow better understanding of how a variable’s influence changed throughout the pandemic. For example, Table 5 shows that the variable indicating white British ethnicity was associated with strong negative effects on the frequency of work trips during Phase 3 of restriction easing, however, this variable had insignificant effects in the Lockdown and Phases 1 to 2 models. Similarly, the gender (male) variable had strong positive effects on the likelihood of frequent work trips during Lockdown, moderate positive effects in Phases 1 to 2, and very strong positive effects in Phase 3, which showed that the greatest disparity in the work patterns of males and females was during Phase 3 of restriction easing.

**Table 5.** Summary of Significant Variables Affecting Work Trips in Lockdown, Phases 1 to 2, and Phase 3 of Restriction Easing

Variable description	Lockdown ZIHOP	Phases 1 to 2 ZIHOPCD	Phase 3 ZIHOP
Splitting function between ordered and zero-state			
Socioeconomic characteristics			
Household social grade (1 if managerial/professional occupation, 0 otherwise)	↓	na	na
Household social grade (1 if semiskilled/unskilled manual occupation or unemployed, 0 otherwise)	↓	na	na
Demographic characteristics			
Health problem or disability (1 if yes, 0 if no)	↓↓	↓↓	↓↓↓
Directly affected by COVID-19 (1 if yes, 0 if no)	na	↑	na
Ordered state			
Socioeconomic characteristics			
Household social grade (1 if managerial/professional occupation, 0 otherwise)	na	↓	↓↓
Household social grade (1 if semiskilled/unskilled manual occupation or unemployed, 0 otherwise)	na	na	↓↓
Demographic characteristics			
Ethnic background (1 if white British background, 0 otherwise)	na	na	↓↓
Gender (1 if male, 0 if female)	↑↑	↑	↑↑↑
Age indicator (1 if over 55, 0 otherwise)	↓↓↓	↓↓	na
Region indicator (1 if major city [Edinburgh or Glasgow], 0 otherwise)	na	na	↑
Behavioral characteristics			
Mode of travel before lockdown (1 if personal vehicle used frequently, 0 if not used frequently)	↑↑	↑	↑↑↑
Heterogeneity in threshold parameters			
Region of Scotland (1 if major city [Edinburgh or Glasgow], 0 otherwise)	↓	↓	na
Age indicator (1 if under 25, 0 otherwise)	na	na	↓

*Note*: ZIHOP = zero-inflated hierarchical ordered probit; ZIHOPCD = zero-inflated hierarchical ordered probit model with correlated disturbances.

Significant variables affecting the splitting function between the ordered- and zero-state allowed insights into the groups who were inherently more likely to belong to the zero-state. The “health problem or disability” variable was the respondent characteristic that was most consistently assigned to the zero-state. Those with a health problem or disability were found to be significantly more likely than those with no disability to belong to the zero-state of trip frequencies in the Lockdown, Phases 1 to 2, and Phase 3 models. This finding is consistent with recent research into commuting preferences, which shows those with a health problem or disability were significantly more likely to telecommute before COVID-19 than those with no disability (*11*). Other prepandemic studies suggest that workers with disabilities may benefit from telecommuting regarding reduced travel times and flexible work schedules, however, this may also lead to these individuals becoming isolated (*26*). Given that telecommuting is expected to be more popular following the pandemic (*10*), future research might assess the pandemic’s effect on the telecommuting intentions of those with a health problem or disability.

Households where the main income earner is employed in a managerial or professional occupation had a significantly negative effect on the probability of the ordered state in the Lockdown model, thus increasing the probability of the zero-state. This could be interpreted as those in managerial or professional occupations being significantly more likely than those belonging to other household social grades to make no work trips, as they seemed more prone to belonging to the zero-state. Given this interpretation, the majority of respondents belonging to the ordered state in the Lockdown model are likely to live in a household where the main income earner is not employed in a managerial or professional occupation. A possible explanation is that the work responsibilities of those in managerial or professional occupations are more easily completed from home, therefore these households were inherently less likely to make work trips during the early stages of the pandemic. This explanation is reinforced by official labor statistics in the United States, which showed that higher income workers (earning more than the 75th percentile) were around six times more likely to be able to telecommute than lower income earners (earning less than the 25th percentile) (*27*). Although these statistics precede the pandemic, it is highly likely that this was still the case during the pandemic in the United States, and possibly also in the UK, therefore this may explain why those in households where the main income earner was employed in a managerial or professional occupation tended to make no work trips. It should be noted that this variable, “household social grade (managerial/professional occupation),” was consistently associated with zero work trips, either affecting the zero-state (Lockdown) or the ordered state (Phases 1 to 2 and Phase 3).

Individuals self-reporting as being directly affected by COVID-19 were also more likely to belong to the ordered state during Phases 1 and 2 of restriction easing. This group of respondents, although possibly quite diverse, might exhibit a tendency to switch to standard travel patterns more easily, especially if they have already contracted COVID-19 and have some degree of immunity against the virus. Interestingly, the concept of perceived “recovery” from COVID-19 may change people’s behavioral intentions, possibly making them more tempted to follow their normal prepandemic patterns (*28*). However, the effect of this variable (“directly affected by COVID-19”) should be interpreted with caution, since the subjective nature of the question might induce unobserved heterogeneity in the responses; this requires further investigation.

Other demographic influences observed in the ordered state were as follows: males were significantly more likely than females to make frequent work trips (six or more) during Lockdown, Phases 1 to 2, and Phase 3; those from a white British ethnic background were significantly more likely to make no work trips during Phase 3, compared with other ethnicities, including any other white, black, Asian or other minority ethnic group; respondents’ region of residence was also influential, such that those who resided in Edinburgh or Glasgow were significantly more likely to make frequent work trips during Phase 3, in comparison to those from other Scottish regions; and respondents over the age of 55 were significantly more likely than other age groups to make no work trips during Lockdown and Phases 1 to 2. As discussed previously, the effect of the gender variable varied across the survey samples. This is likely to be the result of the phased easing of COVID-19 working restrictions. Males are significantly more likely to be employed in manual occupations (e.g., builders, warehouse operatives), many of whom started to return to work during Phases 1 and 2, and even more did so during Phase 3, which may explain the inconsistency of this variable’s effect. Another explanation may be related to gender roles during the pandemic. For example, women were more likely than men to stay at home and provide childcare following the closure of schools (*29*), therefore, it is likely that some women’s ability to work was affected. The disparity in work trip patterns of different ethnic groups in Phase 3 suggests that the work trip patterns of different ethnic groups may be a contributing factor to the disproportionately higher infection risk and mortality rates experienced by black and Asian ethnic minority groups in the UK (*7*). This interpretation is proposed tentatively as the effect may also be partially induced by those from other white ethnic backgrounds (e.g., white European, Romani or Irish).

The increased frequency of work trips among those in Edinburgh and Glasgow is most likely explained by the reopening of nonessential businesses during Phase 3 of restriction easing. It is likely that Scotland’s two most populous cities (Edinburgh and Glasgow) saw increased work trips, relative to other areas in Scotland, because more nonessential businesses (e.g., hospitality venues, gyms, retail outlets) exist in these regions. The tendency for over 55s to make no work trips in Lockdown and Phases 1 to 2 may be linked to COVID-19 risk perceptions, as it is likely that older individuals took extra precautions to avoid contracting COVID-19 in the early stages of the pandemic. This variable had an insignificant effect in Phase 3, which could indicate that some over 55s felt they were able to return to their workplace at this point in the pandemic, and therefore did not have significantly different working patterns from other age groups.

Two socioeconomic characteristics derived from the household social grade variable also significantly influenced the ordered state in the Phase 3 model. Households where the main income earner was employed in a managerial or professional occupation were also significantly more likely to make no work trips compared with other household social grades in Phases 1 to 2. Households where the main income earner was employed in a semiskilled/unskilled occupation or was unemployed were significantly more likely to complete no work trips during Phase 3. The effect of the “household social grade (managerial/professional occupation)” variable corroborates with the previous discussion of this variable—that this group was more likely to telecommute, however, the effect may not have been as pronounced during Phases 1 to 2 and Phase 3 owing to the gradual reopening of some workplaces. The negative effect observed among households where the main income earner was employed in a semiskilled/unskilled occupation or was unemployed, may be partially attributable to this variable including those who were unemployed (an unintended consequence of the survey design), and therefore made no work trips. Given that the categories in “semiskilled/unskilled work” and “unemployed” cannot be distinguished in our dataset, removing responses from households with unemployed main income earners would have required the removal of responses with the main income earner in semiskilled/unskilled works as well. The unemployment rate in Scotland is generally low and during the pandemic employment was protected by a furlough scheme. Therefore, this variable was included in the Phase 3 model, so as not to lose information about households whose main income earner is employed in a semiskilled/unskilled occupation. Aside from this effect, a further explanation may be that the job security of semiskilled/unskilled manual workers has been affected most by the pandemic (*30*), particularly while construction sites and nonessential hospitality businesses were closed. It may be that this demographic also suffered from limited work opportunities as some nonessential workplaces reopened, usually at limited capacity. Those belonging to other household social grades may have had greater capacity to continue working remotely or to return to their workplace, hence the relatively negative effect of the household social grade (semiskilled/unskilled manual occupation/unemployed) variable.

One behavioral characteristic of respondents significantly affected work trip frequencies in the ordered state in all models. Respondents who frequently used a personal vehicle (car or van) before lockdown were significantly more likely to make frequent work trips, in comparison to those who did not use a personal vehicle frequently. A possible explanation may be that those with access to a car were still able to make risk-free work trips, whereas those who did not have access to a car may have perceived public transport to be too risky and instead opted to telecommute. This finding is in line with previous research indicating that as COVID-19 restrictions ease, commuting patterns are expected to follow the trajectory of pre-COVID levels, especially for car users (*31*).

Significant threshold heterogeneity was discovered to be a function of two independent variables: region of Scotland and age. The allowance for threshold heterogeneity resulted in superior GOF for the ZIHOP/ZIHOPCD models, compared with the basic ZIOP models (as shown by the reduced AICs and the statistical significance of LRTs I, II, and III in Table 3). The instances of threshold heterogeneity, which were all discovered for Threshold 1, can be interpreted as follows: a significantly positive (*t*-stat > 1.65) variable causes the threshold to increase, that is, the likelihood of a response in the first interior category (Level 1, 1 to 3 trips) increases relative to the second interior category (Level 2, 4 to 5 trips), whereas a significantly negative variable increases the likelihood of Level 2 relative to Level 1. Two independent variables, region of Scotland (Edinburgh or Glasgow) and age (under 25), significantly affected Threshold 1 across the models. The specific effect of each variable was as follows: respondents who resided in Edinburgh or Glasgow had a higher likelihood than the rest of Scotland of being in Level 1 (1 to 3 weekly work trips) rather than in Level 2 (4 to 5 weekly work trips) in the Lockdown and Phases 1 to 2 models; respondents under 25 had a higher likelihood of being in Level 1 (1 to 3 weekly work trips) compared with other age groups during Phase 3. Those residing in Edinburgh and Glasgow were more likely to make 1 to 3 work trips, as opposed to 4 to 5 trips, which may be the effect of some construction sites and other outdoor workplaces partially reopening during Phases 1 and 2 of restriction easing. Respondents under 25 may have been more likely to make 1 to 3 trips during Phase 3 because of their occupation, for example, this variable is likely to include students who may have part-time occupations in nonessential businesses, some of which reopened in Phase 3. It should be noted that the variables that were found to capture threshold heterogeneity were first tested as regular independent variables, however their effect was statistically insignificant.

As mentioned previously, the disturbance terms between the binary- and ordered probit components of the Phases 1 to 2 model were found to be correlated, as evidenced through the statistically significant correlation coefficient reported in Table 3. For this case, a bivariate standard normal distribution was used for the calculation of the ordered likelihoods, allowing the disturbance terms to be unrestrictedly correlated (*22*). The coefficient was strong in magnitude (−0.763) showing the presence of common unobserved characteristics influencing the likelihoods for both underlying states. Furthermore, the negative sign demonstrated that these characteristics may affect the state splitting function and the trip frequencies differently. For example, unobserved factors, which increase the likelihood of a respondent belonging to the ordered state, may at the same time decrease the likelihood of frequent work trips, and vice versa. It is difficult to tell exactly what these unobserved factors might be, however, this does provide another interesting question for further research. It should be noted that the correlation of disturbance terms was also tested in the other two models, but were statistically insignificant.

## Conclusions

This study used survey data to show how the work trip patterns of Scottish residents varied throughout the COVID-19 lockdown and subsequent phases of restriction easing. During Lockdown, a small proportion of respondents (∼15%) made at least one weekly work trip, whereas the remaining 85% made no work trips. There was little change during Phases 1 to 2 of the Scottish Government’s COVID-19 route map, as 87% made no weekly work trips. It is likely that those making work trips during Lockdown and Phases 1 and 2 were almost exclusively key workers (e.g., healthcare, emergency services). Those who made no work trips during Lockdown and Phases 1 and 2 were likely to have telecommuted if their occupation permitted this, or if not, were eligible for the UK Government’s furlough scheme. The largest shift in behavior was during Phase 3 of restriction easing, which saw ∼23% of respondents make at least 1 weekly work trip, whereas the remaining 77% made no trips. The increase in work trips made during Phase 3 is likely to be the direct result of nonessential businesses reopening, as many of those employed in the hospitality, leisure, or retail sectors will have returned to work at this point.

The ZIHOP models provided valuable insights into Scottish residents’ work trips throughout the pandemic, revealing significant inequalities between different sociodemographic groups. Those from a white British ethnic background were found to be significantly more likely than other ethnicities (ethnic minority groups, including other white ethnicities) to have made no weekly work trips during Phase 3; whereas those who lived in households where the main income earner was employed in a managerial/professional occupation and those with a health problem or disability, were both significantly more likely to have made no weekly work trips across all models. Respondents with a health problem or disability belonged to the zero-state in all three models, showing that this demographic was more likely to make no work trips. The finding that those from a white British ethnic background were significantly more likely to make no work trips during the later stages of the pandemic provides evidence of the link between ethnicity, work trip patterns, and the risk of COVID-19 infection. It is possible that ethnic disparities in the work trips of Scottish residents were a contributing factor to the disproportionate infection and mortality rates experienced by black and Asian ethnic minority groups (*7*). However, one caveat is that this variable’s effect on frequency of work trips may have been partially induced by the remaining control group (any other white background).

The finding that social AB households made significantly fewer work trips than other social grades is almost certainly related to the nature of their occupations. This finding highlights that households where the main income earner was employed in a managerial/professional occupation benefited from low-risk work throughout the pandemic (in relation to the COVID-19 contraction risk associated with continued commuting and workplace attendance [*6*]). Those with a health problem or disability were significantly more likely to make no weekly work trips during lockdown and were inherently more likely to make no work trips in the remaining periods. This finding suggests that those with a health problem or disability were significantly more likely to telecommute during the pandemic, a trend that may persist even when COVID-19 no longer poses a risk.

Several limitations of this study should be noted. Firstly, the survey data lack attitudinal variables; for example, COVID-19 risk perceptions, telecommuting preferences, and social norms may also have affected work trip patterns. Secondly, the household social grade (semiskilled/unskilled manual occupation or unemployed) variable combined those in semiskilled/unskilled work and those who were unemployed. As a result, this variable's effect is likely to have been unduly inflated. Finally, given that survey respondents were selected from landline records, the sample excluded Scottish residents who do not own a landline. As of 2021 in Scotland, 34% of households did not own a landline.

Future research should focus on the sociodemographic inequalities in work trip patterns identified across all models. This is a cause for concern, as our results indicated that socioeconomic factors played a significant role in dictating work trips patterns throughout the COVID-19 pandemic. Attention should also be given to the future working patterns of those with a health problem or disability, who were significantly less likely to make work trips throughout the pandemic. For those with a health problem or disability, working from home has the potential to improve or worsen social inequalities, for example, an individual with a physical disability may benefit from reduced commuting but this may also lead to social isolation, particularly if most workplaces revert to in-person work. Given that Transport Scotland’s *National Transport Strategy* (*32*) prioritizes the reduction of social inequalities and the improvement of public health and wellbeing, we recommend that Transport Scotland considers further investigation of the role COVID-19 has played in exacerbating social, economic, and health inequalities.

## Supplemental Material

sj-docx-1-trr-10.1177_03611981221119192 – Supplemental material for Who is More Likely (Not) to Make Home-Based Work Trips During the COVID-19 Pandemic? The Case of Scotland Click here for additional data file. Supplemental material, sj-docx-1-trr-10.1177_03611981221119192 for Who is More Likely (Not) to Make Home-Based Work Trips During the COVID-19 Pandemic? The Case of Scotland by Torran Semple, Grigorios Fountas and Achille Fonzone in Transportation Research Record
