# Supplementary material for: Who is More Likely (Not) to Make Home-Based Work Trips During the COVID-19 Pandemic? The Case of Scotland
Source: Transp Res Rec. 2022 Sep 20;2677(4):904–16. doi: 10.1177/03611981221119192 (PMC9490396; doi:10.1177/03611981221119192)
Supplement: sj-docx-1-trr-10.1177_03611981221119192 – Supplemental material for Who is More Likely (Not) to Make Home-Based Work Trips During the COVID-19 Pandemic? The Case of Scotland [file sj-docx-1-trr-10.1177_03611981221119192.docx]

**APPENDIX**

Table A1 – Independent variables available for modelling

| **Variable No.** | **Variable Description** |
| --- | --- |
| 1 | **Gender**: Male, Female, Non-binary |
| 2 | **Age**: Under 16, 16-24, 25-34, 35-44, 45-54, 55-64, 65-74, 75-84, 85+ |
| 3 | **Ethnic background**: White British, Any other White background, Any mixed background, Indian, Pakistani, Bangladeshi, Chinese, Any other Asian background, Caribbean, African, Any other Black background, Any other background |
| 4 | **Region of Scotland**: Argyll & Bute, Ayrshire & Arran, Edinburgh and South East Scotland, Forth Valley, Glasgow City, Highlands and Islands, North East Scotland, Scottish Borders, South West Scotland, Tay Cities Region |
| 5 | **Health problem or disability that limits day-to-day activities**: Yes (a lot), Yes (a little), No |
| 6 | **Employment status (of the household’s main income earner)**: Higher managerial, administrative, or professional; Intermediate managerial, administrative or professional; Supervisory, clerical, junior managerial, administrative or professional; Skilled manual workers; Semi and unskilled manual worker; Unemployed/currently not working; Housewife/husband; State pensioner/retired; Student |
| 7 | **Household social grade (based on the employment status of the household’s main income earner)**: AB (higher/intermediate managerial, administrative or professional occupations), C1 (supervisory, clerical, junior managerial, administrative or professional, and students), C2 (skilled manual workers), DE (semi/unskilled manual worker or unemployed) |
| 8 | **Current working situation**: Any form of self-employment, Any form of employment (not furloughed), Currently employed but furloughed, Full-time education, Retired, Unemployed, Long-term sick/disabled/looking after household |
| 9 | **Directly affected by COVID-19**: Yes, No |
| 10 | **Most frequently used modes of travel before COVID-19**: Public transport (bus, train or tram), Personal vehicle (car, van or taxi), Active travel (on-foot, by wheelchair or by bicycle) |
| 11 | **Mode of travel before and during COVID-19**: E.g. Public transport frequently used before COVID-19 but used less during COVID-19 |
